# Arsenic Resistance and Biosorption by Isolated Rhizobacteria from the Roots of *Ludwigia octovalvis*

**DOI:** 10.1155/2018/3101498

**Published:** 2018-12-30

**Authors:** Harmin Sulistiyaning Titah, Siti Rozaimah Sheikh Abdullah, Mushrifah Idris, Nurina Anuar, Hassan Basri, Muhammad Mukhlisin, Bieby Voijant Tangahu, Ipung Fitri Purwanti, Setyo Budi Kurniawan

**Affiliations:** ^1^Department of Environmental Engineering, Faculty of Civil, Environmental and Geo Engineering, Institut Teknologi Sepuluh Nopember (ITS), Kampus ITS Keputih, Sukolilo, 60111 Surabaya, Indonesia; ^2^Department of Chemical and Prosess Engineering, Faculty of Engineering and Built Environment, Universiti Kebangsaan Malaysia, 43600 UKM Bangi, Selangor, Malaysia; ^3^Tasik Chini Research Centre, Faculty of Science and Technology, Universiti Kebangsaan Malaysia, 43600 UKM Bangi, Selangor, Malaysia; ^4^Department of Civil and Structural Engineering, Faculty of Engineering and Built Environment, Universiti Kebangsaan Malaysia, 43600 UKM Bangi, Selangor, Malaysia; ^5^Department of Civil Engineering, Politeknik Negeri Semarang, Semarang, Indonesia

## Abstract

Certain rhizobacteria can be applied to remove arsenic in the environment through bioremediation or phytoremediation. This study determines the minimum inhibitory concentration (MIC) of arsenic on identified rhizobacteria that were isolated from the roots of *Ludwigia octovalvis* (Jacq.) Raven. The arsenic biosorption capability of the was also analyzed. Among the 10 isolated rhizobacteria, five were Gram-positive (*Arthrobacter globiformis*, *Bacillus megaterium, Bacillus cereus, Bacillus pumilus*, and *Staphylococcus lentus*), and five were Gram-negative (*Enterobacter asburiae, Sphingomonas paucimobilis, Pantoea* spp.*, Rhizobium rhizogenes*, and *Rhizobium radiobacter*). *R. radiobacter* showed the highest MIC of >1,500 mg/L of arsenic. All the rhizobacteria were capable of absorbing arsenic, and *S. paucimobilis* showed the highest arsenic biosorption capability (146.4 ± 23.4 mg/g dry cell weight). Kinetic rate analysis showed that *B. cereus* followed the pore diffusion model (*R*^2^ = 0.86), *E. asburiae* followed the pseudo-first-order kinetic model (*R*^2^ = 0.99), and *R. rhizogenes* followed the pseudo-second-order kinetic model (*R*^2^ = 0.93). The identified rhizobacteria differ in their mechanism of arsenic biosorption, arsenic biosorption capability, and kinetic models in arsenic biosorption.

## 1. Introduction

Currently, most research on phytoremediation has emphasized on the physiological mechanisms of plant transport and tolerance, as well as the plant's storage of metal. A few information is available on processes in the hyperaccumulator plant phytoextraction of metals [1]. Rhizobacteria are usually present in soils naturally, despite having high amounts of metals.

Many bacterial strains contain the genetic determinants of resistance to heavy metals such as arsenic, bismuth, cadmium, chromium, lead, mercury, nickel, silver, and others [2]. The bioavailable and extractable forms of arsenic significantly inhibit the soil microbial community, although bioavailable arsenic exhibits better inhibitory effect than total arsenic [3]. Some bacteria are resistant to arsenic due to their ability to remove it from their surroundings [4]. Many Gram-negative and Gram-positive bacteria have detoxification mechanisms [5]. Bacteria have various ways of coping with high levels of arsenic, including reduced uptake, methylation following the reduction of arsenate to arsenite, the adsorption of negatively charged arsenic ions by the oppositely charged amino groups in bacterial cell walls, sequestration by a range of cysteine-rich peptides, chelation, compartmentalisation, exclusion, immobilization, and dissimilatory arsenate respiration [5, 6]. Although arsenic is toxic to many bacteria, metal-accumulating bacteria are often found among metal-resistant bacteria [4]. Nanda and Abraham [2] found that the toxicity of arsenic is higher than chromium, magnesium, and copper (As > Cr > Mg > Cu). Arsenate is toxic to bacteria, because it is analogous to phosphate and can inhibit enzymes such as kinases [7].

Rhizobacteria that are capable of aggressively colonising plant roots and promoting plant growth are generally known as plant growth-promoting rhizobacteria (PGPR) [8]. PGPR can invade the interior of the cells and survive inside (intracellular PGPR, such as nodule bacteria), or remain outside the plant cells (extracellular PGPR, such as *Bacillus*, *Pseudomonas*, and *Azotobacter*). PGPR such as *Agrobacterium* (*Rhizobium*)*, Alcaligenes* (*Ralstonia*)*, Arthrobacter, Azospirillum, Azotobacter, Bacillus, Burkholderia, Serratia*, and *Pseudomonas* are interesting organisms for the metal extraction by plants, because they increase the rate of both metal and biomass accumulation by plants [9]. Contaminated soils are often poor in nutrients or sometimes nutrient-deficient due to the loss of useful microbes. However, such soils can be made nutrient-rich by applying metal-tolerant microbes, especially PGPR, which not only provide essential nutrients to the plants properties allow plants to remove heavy metals, which can then be useful in agricultural production or the phytoremediation of contaminated soil [11, 12]. Therefore, it is advisable for growers to inoculate plants with growing in the contaminated sites but also play a major role in detoxifying heavy metals [8, 10]. These rhizobacterial microbes to increase plant biomass and stabilise, revegetate, and restore heavy metal-contaminated soils [9].

The interactions of rhizobacteria and plants in remediating arsenic have been studied. According to Titah et al. [13]; the application of a six-rhizobacterial consortium alleviated the toxic effects of arsenic in *Ludwigia octovalvis* and increased the plant biomass. According to Nie et al. [14], canola plants inoculated with *Enterobacter cloacae* have grown to a significantly higher extent than noninoculated canola plants due to the presence of arsenic. Glick et al. [15] reported the increase in shoot biomass and arsenic concentration in the shoots of *Helianthus annuus* after inoculation with *Pseudomonas fluorescens*.

*L. octovalvis* is a plant species that can survive at a crude oil-contaminated site [16] and could uptake and accumulate arsenic in its tissues [17]. This study aimed to determine the level of arsenic (as arsenate) resistance of the identified rhizobacteria that were isolated from the roots of *L. octovalvis* grown in the greenhouse and contaminated land in Malacca, Malaysia. It also aimed at determining their arsenic biosorption capability.

## 2. Materials and Methods

### 2.1. Epiphyte Rhizobacterial Isolation from the Root of *L. octovalvis*

The detailed steps of rhizobacterial isolation was described by Titah et al. [18].

### 2.2. Identification of Rhizobacteria

Rhizobacterial identification was conducted using two biochemical methods: Biolog GEN III microbial identification system (Biolog, Inc, USA) and Vitek2 Compact System (Biomerieux, USA). The detailed steps of rhizobacteria identification was described by Titah et al. [18].

### 2.3. Determination of the Minimum Inhibitory Concentration (MIC) of Arsenic

The test was conducted using the modified method of Guo et al. [19]. The MIC of arsenate for each rhizobacterium species was determined in three replicates of sterile phosphate buffer saline (PBS) solution with a serial concentration of 0 (control), 250, 500, 750, and 1,000 mg/L of arsenic in arsenate form (sodium arsenate dibasic heptahydrate (AsHNa_2_O_4_·7H_2_O)) (FlukaChemika, Switzerland). Two different controls were used for this method: a PBS solution with bacteria but without arsenate (negative control) and another PBS solution with arsenate but without bacteria (positive control). Based on the study of Harley and Prescott [20], 10 × PBS stock solution contained 80 g NaCl (Merck, Germany), 2 g KCl (R&M Chemicals, India), 11 g Na_2_HPO_4_·7H_2_O (R&M Chemicals, India), and 2 g KH_2_PO_4_ (R&M Chemicals, India). MIC determination was carried out in a 250 mL Erlenmeyer flask with 50 mL working volume. The pH of arsenate in sterile PBS solution was measured using Accument Basic AB 15 pH meter (Fisher Scientific, USA). The average pH readings were 7.3, 7.4, 7.5, 7.6, and 7.7 in the arsenate concentrations of 0, 250, 500, 750, and 1,000 mg/L, respectively. The cultures were incubated using an incubator shaker (Protech, Model SI-100D, Malaysia) at 37°C and 150 rpm. Bacterial growth was determined at an absorbance of 550 nm for 0, 3, 6, 19, and 24 h.

### 2.4. Arsenic Biosorption Experiment Using Identified Rhizobacteria in Batch System

An arsenic biosorption experiment was conducted using a modified published method [21–23]. Selected rhizobacteria were inoculated onto Trypticase (Tryptic) soy agar (TSA) media without arsenate for 24 h. After that, the selected rhizobacteria were inoculated into sterile Tryptic soy broth (TSB). The cell suspension of the selected rhizobacteria was prepared by harvesting the cells at the middle of the logarithmic phase based on the typical of growth rate graph for the selected rhizobacteria. At this time, the optical density (OD) at 550 nm was 1.0. The cells were harvested through centrifugation (Eppendorf centrifuge 5804, Germany) at 4,000 rpm for 15 min. The obtained pellet was then washed twice by using 8.5 g NaCl/1,000 mL solution.

The biosorption of arsenic was then tested in a 250 mL Erlenmeyer flask. The rhizobacterial cell suspension at 20% (*v*/*v*) or 10 mg dry cell weight (DCW) was added with 50 mL of sterile PBS solution, which had an arsenic concentration of 100 mg/L by diluting sodium arsenate dibasic heptahydrate (AsHNa_2_O_4_·7H_2_0) (Fluka Chemika, Switzerland). The colonyforming unit (CFU) of the initial rhizobacteria was approximately 1.4 × 10^9^ CFU/mL. A pH of 7.3 was measured for 100 mg/L arsenate in sterile PBS solution by using Accument Basic AB 15 pH meter (Fisher Scientific, USA).

All samples were tested in triplicates. All the cultures were incubated using an incubator shaker (Protech, Model SI-100D, Malaysia) at 37°C and 150 rpm. Samples were harvested at 0, 2, 6, 17, and 24 h. The OD was measured at 550 nm at each sampling by using Genesys 10 UV (Thermo Spectronic, USA). Finally, these rhizobacterial suspensions were serially diluted, and 0.1 mL of this sample was spread onto TSA. The number of colonies grown was counted in CFU/mL. The dry weight of the rhizobacteria cells was determined by filtering the suspension culture through a vacuum filter by using 0.2 *μ*m membrane filter paper (Whatman, Germany). The DCW was determined after overnight drying at 105°C [24].

Ten mL of each culture was taken at each sampling time, and the supernatant and pellet were separated through centrifugation (Eppendorf Centrifuge 5804, Germany) at 10,000 rpm for 15 min. The residual amount of the total arsenic present in the supernatant was stored at −80°C [6] before inductively coupled plasma-optical emission spectrometer (ICP–OES) analysis by using Optima 7300DV PerkinElmer (USA) for the total arsenic. The cell pellet was dried at room temperature for 2 days [25]. Then, 1 mL of 69% HNO_3_, 1 mL of 30% H_2_O_2_, and 3 mL of sterile pure water were added to the cell pellet for 24 h. The cell samples were stored at −20°C [24], and the arsenic concentration of the cells was determined using ICP–OES Optima 7300DV PerkinElmer (USA).

### 2.5. Stastitical Analysis

All statistical tests were performed using SPSS Version 17.0 (IBM, USA). Two-way analysis of variance (ANOVA) at 95% confidence level (*p* < 0.05) was used to evaluate significant changes in the total arsenic concentration in the supernatant and the biosorption capability of the rhizobacteria. Correlation analysis was performed to determine the relationship between the concentration of arsenic in the supernatant and the capability of arsenic biosorption by rhizobacteria by using the Pearson correlation.

### 2.6. Transmission Electron Microscopy (TEM) Analysis

TEM images of selected rhizobacterium, which had the highest biosorption capability, were obtained after exposure to arsenate for 48 h. The arsenic concentration was set according to the results of the MIC experiment. TEM was performed to determine the crosswise and longitudinal structure of the cell after exposure to arsenic compared with the unexposed cells. Objective analysis of spectral elements was conducted to determine the arsenic content in the cells of the rhizobacterium, which was conducted using a TEM equipment model Philips CM 12 (Netherlands).

### 2.7. Biosorption Kinetic Models

In the batch experiments, kinetic studies were performed to determine the contact time of the adsorbent with the adsorbate and evaluate the reaction coefficients. To analyze the uptake kinetics, many models such as pseudo-first-order, pseudo-second-order, pore diffusion, and Elovich equation can be used. The pseudo-first-order equation of Lagergren is based on the solid capacity [26], whereas the pseudo-second-order reaction model is based on solid-phase adsorption and implies that chemisorption is the rate-controlling step [27]. The pore diffusion model is based on intraparticle diffusion processes [28], whereas the Elovich equation model is based on the best fit adsorption mechanisms.

#### 2.7.1. Pseudo-First-Order Model

The pseudo-first-order equation is generally expressed as follows:

(1) $$\begin{array}{c} \frac{dq_{t}}{q_{t}}=k_{1}\left(q_{\text{e}}-q_{t}\right), \end{array}$$

where *q*_e_ and *q*_*t*_ are the adsorption capacities at equilibrium and at time *t* (mg/g biomass) and *k*_1_ is the rate constant of the pseudo-first-order adsorption (h). A linear form of the pseudo-first-order model was described by Lagergren. By integrating the equation and applying boundary conditions of *t*=0 to *t*=*t*, and *q*_e_=0 to *q*_*t*_=*q* at *t*, it becomes

(2) $$\begin{array}{c} \log\left(q_{\text{e}}-q_{t}\right)=\log\left(q_{\text{e}}\right)-\frac{k_{1}}{2.303}t. \end{array}$$

A plot of log(*q*_e_ − *q*_*t*_) against *t* gives −(*k*_1_/2.303) as the slope and log(*q*_e_) as the intercept.

#### 2.7.2. Pseudo-Second-Order Model

The pseudo-second-order model is used to describe the sorption kinetics [27]. The model assumes that the rate-limiting step may be chemical sorption (or chemisorption) involving valence forces through sharing or the exchange of electrons between the sorbent and sorbate [29]. This model is represented by the following equation [30]:

(3) $$\begin{array}{c} \frac{qdq_{t}}{d_{t}}=k_{2}{\left(q_{\text{eq}}-q_{t}\right)}^{2}, \end{array}$$

where *k*_2_ is the rate constant of pseudo-second-order adsorption (mg/g biomass/h). By applying the boundary conditions *t*=0 to *t*=*t*, and *q*_e_=0 to *q*_*t*_=*q* at *t*, the integrated form of the equation becomes

(4) $$\begin{array}{c} \frac{1}{\left(q_{\text{eq}}-q_{t}\right)}=\frac{1}{q_{\text{eq}}}+k_{2}t, \end{array}$$

which is the integrated rate law for a pseudo-second-order reaction, which can be rearranged to obtain the following linear form:

(5) $$\begin{array}{c} \frac{t}{q_{t}}=\frac{1}{\left(k_{2}q_{\text{eq}}^{2}\right)}+\frac{1}{q_{\text{eq}}}t. \end{array}$$

A plot of *t*/*q*_*t*_ versus *t* should provide a linear relationship for the applicability of the second-order kinetics. The rate constant (*k*_2_) and adsorption at equilibrium (*q*_eq_) can be obtained from the intercept and slope, respectively.

#### 2.7.3. Pore Diffusion Order Model

In most adsorption processes, the uptake varies almost proportionally with *t*^1/2^:

(6) $$\begin{array}{c} q_{t}=K_{\text{d}}t^{1/2}, \end{array}$$

where *K*_d_ (mg/g/min^1/2^) is the diffusion rate constant and *q*_*t*_ is the amount of adsorbate adsorbed at *t*. A plot of *q*_*t*_ against *t*^1/2^ yields a slope of *K*_d_. The Weber–Morris plot versus *t*^1/2^ should be a straight line with a slope *K*_d_ and intercept of *C* when adsorption mechanisms follow the intraparticle diffusion processes [31]. According to Weber and Morris [28], q sorption processes will be controlled by the slowest of the rate-limiting steps.

#### 2.7.4. Elovich Equation Model

The Elovich equation has been commonly used in determining the kinetics of chemisorption on gases and solids [29]. However, some researchers have applied this model to solid-liquid sorption systems, especially in the sorption of heavy metals [32, 33]. The Elovich equation is as follows:

(7) $$\begin{array}{c} q_{t}=\left(\frac{1}{\beta }\right)\ln\left(\alpha \beta \right)+\left(\frac{1}{\beta }\right)\ln t, \end{array}$$

where *q*_*t*_ is the amount of adsorbate adsorbed per unit *t* and *α* and *β* are the Elovich constants, *β* is the initial adsorption rate of the Elovich equation (mg/g·h), and *α* is related to the extent of surface coverage (mg/g) and activation energy for chemisorption [34]. A plot of *q*_*t*_ against ln*t* yields (1/*β*) as the slope and (1/*β*) ln(*αβ*) as the intercept.

## 3. Results and Discussion

### 3.1. MIC

Ten rhizobacteria from the roots of *L. octovalvis* tested for their resistance to arsenate include *Arthrobacter globiformis*, *Bacillus megaterium, Bacillus cereus, Bacillus pumilus, Staphylococcus lentus, Enterobacter asburiae, Sphingomonas paucimobilis, Pantoea* spp.*, Rhizobium rhizogenes*, *and Rhizobium radiobacter*.  Table 1 shows the summary of the MIC of arsenate for all rhizobacteria. *R. radiobacter* had the highest MIC (>1500 mg/L), indicating that it was the most resistant rhizobacterium to arsenic (in the arsenate form). *R. rhizogenes* had an MIC of arsenate of 750 mg/L. The two *Rhizobium* species were isolated from the sand spiked with the highest arsenate concentration (80 mg/kg) [35]. The growth of *R. rhizogenes* and *Sphingomonas paucimobilis* was inhibited at an arsenate level of 750 mg/L. The MIC of arsenate of *A. globiformis, B. pumilus, Staphylococcus lentus,* and *E. Asburiae* was 500 mg/L, while that of *B. megaterium*, *B. cereus*, *and Pantoea* spp. was 250 mg/L.

The arsenate resistance of *A. globiformis* was higher than *B. megaterium* and *B. cereus*, which agree with the findings of Achour et al. [36]. According to Achour et al. [36], the MIC of arsenate to *Arthrobacter* sp. on a solid medium (Luria–Bertani (LB) agar) was 160 mM, whereas that of *Bacillus* sp. was 50 mM. Another study reported the MIC of arsenate for *Arthrobacter* sp. and *Bacillus* sp. on LB agar was above 75 mM [37].

The MIC for arsenate of Gram-positive rhizobacteria was lower than that of Gram-negative rhizobacteria. This finding indicates that the Gram-negative rhizobacteria are more inhibited by arsenate or are less resistant to arsenate than Gram-positive rhizobacteria. This condition may be caused by the differences in the cell wall structure between Gram-negative and Gram-positive rhizobacteria. The cell walls of Gram-negative rhizobacteria are more complex than those of Gram-positive rhizobacteria. The cell walls of Gram-negative rhizobacteria had outer layers of membrane (OM), which selectively protects rhizobacteria from harmful substances such as antibiotics, heavy metals, and other toxic chemicals [38]. OM contains protein, fat, and lipopolysaccharide. A study reported by Anyanwu and Ugwu [39] shows that 67% of the 12 species were Gram-negative bacteria that were more resistant to arsenic exposure than Gram-positive bacteria.

### 3.2. Biosorption Capability of the Identified Rhizobacteria

**Figure 1:**
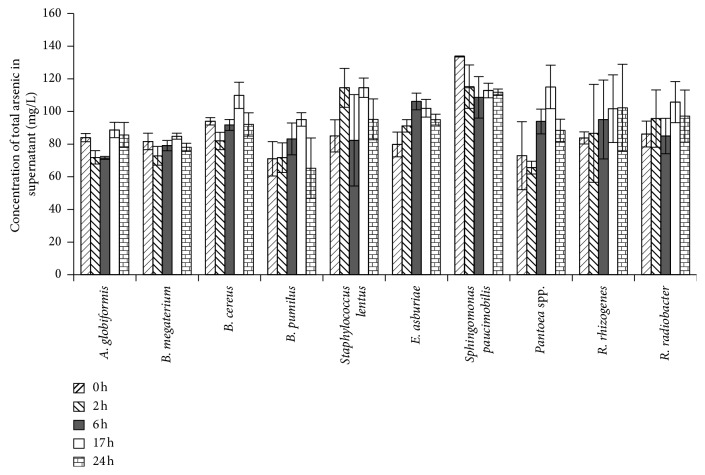
Concentration of arsenic in the supernatant throughout the 24 h of exposure with individual rhizobacteria. Vertical bars indicate SD of triplicates.

Figure 1 shows the concentrations of total arsenic in the supernatant throughout the biosorption test. The total arsenic concentrations decreased within the first 2 h, increased in *A. globiformis, B. megaterium,* and *B. cereus* for 17 h, and decreased in the supernatant of the three rhizobacteria at the end of the exposure (24 h). The increase in total arsenic concentrations in the supernatant might be due to the conversion of arsenate to arsenite by the rhizobacteria, which is later pumped out of the cell [5]. Botes et al. [6] reported that hyper-resistant bacteria can take up 50%–100% of arsenate and export approximately 15%–25% as arsenite.

*B. pumilus* showed a different trend. Its total arsenic concentration in the supernatant increased up to 17 h and then decreased until the end of the exposure. *Staphylococcus lentus* also showed a different trend with increasing and decreasing arsenic concentration in the supernatant from 2 h to 24 h. In *E. asburiae* and *Pantoea* spp., the total arsenic concentration in the supernatant increased up to 6 h and then decreased. The arsenic concentration in the supernatant for *Sphingomonas paucimobilis* had the highest decrease at 2 h, whereas those for *R. rhizogenes* and *R. radiobacter* increased until the end of the exposure (24 hour). The arsenic removal after 24 h of the rhizobacteria can be arranged as follows: *Sphingomonas paucimobilis* > *B. pumilus* > *B megaterium* > *B. cereus* > *A. globiformis* > *Staphylococcus lentus* > *R. radiobacter* > *E. asburiae* > *Pantoea* spp. > *R. rhizogenes*.

Arsenate enters the bacterial cell wall through a fast, unspecific, and constitutive uptake system for phosphate [40]. Arsenate detoxification involves its reduction to arsenite by arsenate reductase, prior to its efflux through a membrane potential driven pump controlled by *trans*-acting repressor (ArsR). The ArsR protein contains a very specific binding site towards arsenite and can discriminate effectively against phosphate, sulphate, cobalt, and cadmium. Although ArsR is specific for arsenite, the removal of arsenate may occur through the initial conversion of arsenate into arsenite by arsenate reductase and subsequent sequestration by ArsR [40].

The results of ANOVA show that the total arsenic concentration in the supernatant was significantly different depending on the rhizobacteria species and the exposure time (*p* < 0.05). The total arsenic concentration in the supernatant for *Sphingomonas paucimobilis* was significantly different (*p* < 0.05) compared with those of the other nine rhizobacteria at 0 h. However, at 24 h, the total arsenic concentration in the supernatant for *Sphingomonas paucimobilis* was significantly different (*p* < 0.05) than those of the seven other rhizobacteria but was not different (*p* > 0.05) from *R. rhizogenes* and *R. radiobacter*.

**Figure 2:**
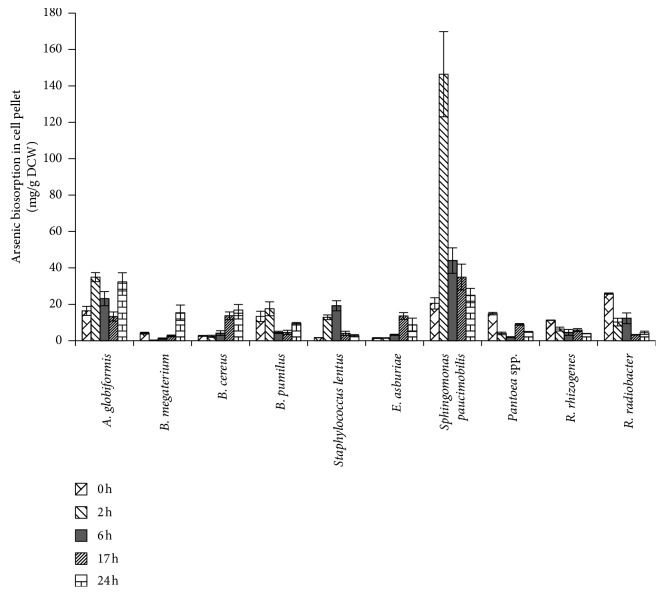
Arsenic biosorption capability of *A. globiformis*, *B. megaterium, B. cereus, B. pumilus*, *Staphylococcus lentus*, *E. asburiae*, *Sphingomonas paucimobilis, Pantoea* spp., *R. rhizogenes,* and *R. radiobacter*. Vertical bars indicate SD of the triplicates.

Figure 2 shows that each rhizobacterium species had total arsenic concentrations at different cells, indicating that the arsenic biosorption capabilities of each species differ. *Sphingomonas paucimobilis* had the highest arsenic biosorption (146.4 ± 23.4 mg/g DCW) at 2 h. The maximum arsenic biosorption of *B. cereus*, *B. megaterium,* and *B. pumilus* occurred at 24 h with 16.8 ± 4.2, 15.4 ± 3.1, and 9.4 ± 0.6 mg/g DCW, respectively. According to Shakya et al. [41], the accumulation of arsenic by *B. cereus* decreased from 24 to 96 h of exposure. Another study reported that the maximum arsenic accumulation of *Bacillus* spp. strain DJ-1, which was observed during the stationary phase of growth, was 9.8 ± 0.5 mg/g DCW [42]. The arsenic biosorption of *A. globiformis* at 24 h was 32.2 ± 5.0 mg/g DCW. The arsenic biosorption of *Staphylococcus lentus* increased up to 6 h (19.2 ± 2.8 mg/g DCW) and then decreased up to 24 h (2.8 ± 0.6 mg/g DCW). The arsenic biosorption of *Pantoea* spp. decreased up to 6 h (2.0 ± 0.2 mg/g DCW), increased, and then decreased to 24 h (4.8 ± 0.3 mg/g cell dry weight). The arsenic biosorption of *E. asburiae* increased up to 17 h (13.5 ± 1.9 mg/g DCW) and then decreased at 24 h (8.7 ± 3.7 mg/g DCW). The arsenic biosorption of *R. rhizogenes* and *R. radiobacter* declined with arsenic levels of 11.2 ± 0.1 to 3.9 ± 0.1 and 25.9 ± 0.4 to 4.3 ± 0.9 mg/g DCW, respectively. The average arsenic biosorption capability after 24 h of the rhizobacteria can be arranged as follows: *Sphingomonas paucimobilis* > *A. globiformis* > *R. radiobacter* > *B. pumilus* > *Staphylococcus lentus* > *B. cereus* > *Pantoea* spp. > *R. rhizogenes* > *E. asburiae* > *B. megaterium*.

ANOVA shows that the arsenic biosorption capabilities differed significantly among rhizobacteria species (*p* < 0.05). However, the exposure time did not significantly differ (*p* > 0.05). The LSD analysis on the capability of arsenic biosorption indicates that the arsenic biosorption capacity of *Sphingomonas paucimobilis* and *A. globiformis* was significantly different (*p* < 0.05) compared with the other eight rhizobacteria.

Correlation analysis was conducted to determine the relationship between the concentration of arsenic in the supernatant, and the arsenic biosorption ability of the rhizobacteria was tested using the Pearson correlation. Results show a correlation coefficient of −0.3 at the 0.01 confidence level. The negative value indicates an inverse correlation relationship. As the concentration of arsenic in the supernatant decreased, the ability of rhizobacteria to take up arsenic increased.

### 3.3. TEM Analysis

**Figure 3:**
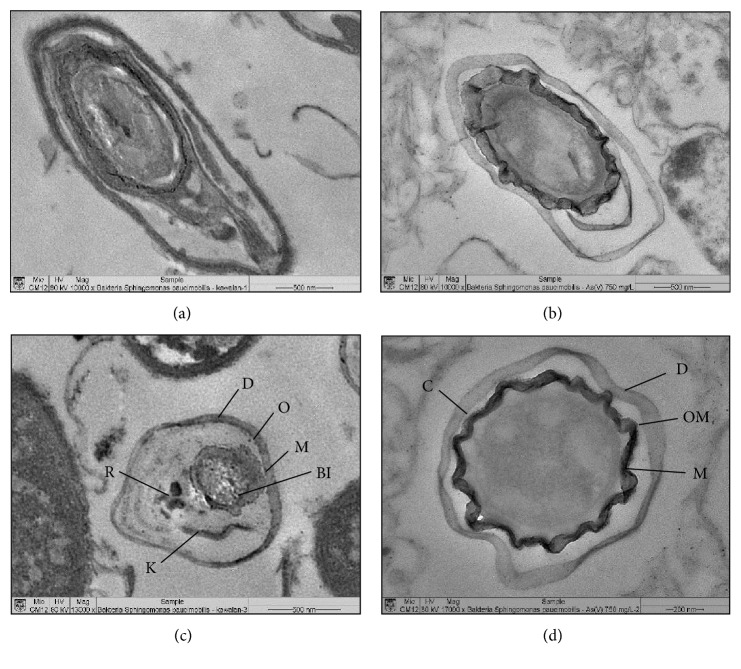
TEM analysis: (a) control 10,000×, (b) 750 mg/L arsenate 10,000×, (c) control 13,000×, and (d) 750 mg/L arsenate 17,000×.

The TEM analysis shows a significant difference in *Sphingomonas paucimobilis* cells exposed to an arsenate concentration of 750 mg/L and the unexposed (control) cells.  Figure 3 shows the results of the TEM analysis of *Sphingomonas paucimobilis* cells with elongated (Figures 3(a) and 3(b)) and transverse pieces (Figures 3(c) and 3(d)). No damage was observed to the unexposed cells of *Sphingomonas paucimobilis*.  Figure 3(c) shows that the cell wall (D), OM, boasts a plasma cell (M), cell cytoplasm and its interior, ribosomes (R), chromosomes (K), and inclusion bodies (BI) in the control treatment. Cells exposed to arsenic (in arsenate form, AsO_4_^−3^ or As(V)) had thickened and wrinkled both the cell wall and the cell plasma membrane. *Sphingomonas paucimobilis* cells grown at an arsenate concentration of 750 mg/L were smaller than those grown under the control medium (Figures 3(a) and 3(b)). The presence of a capsule (C) could be associated with a method of cell protection against arsenate exposure.

### 3.4. Biosorption Kinetic Model for Arsenic

For the biosorption kinetic studies, various kinetic equations were tested on the data to determine their fitness.  Table 2 shows the summary of the four kinetic models for ten identified rhizobacteria.

**Figure 4:**
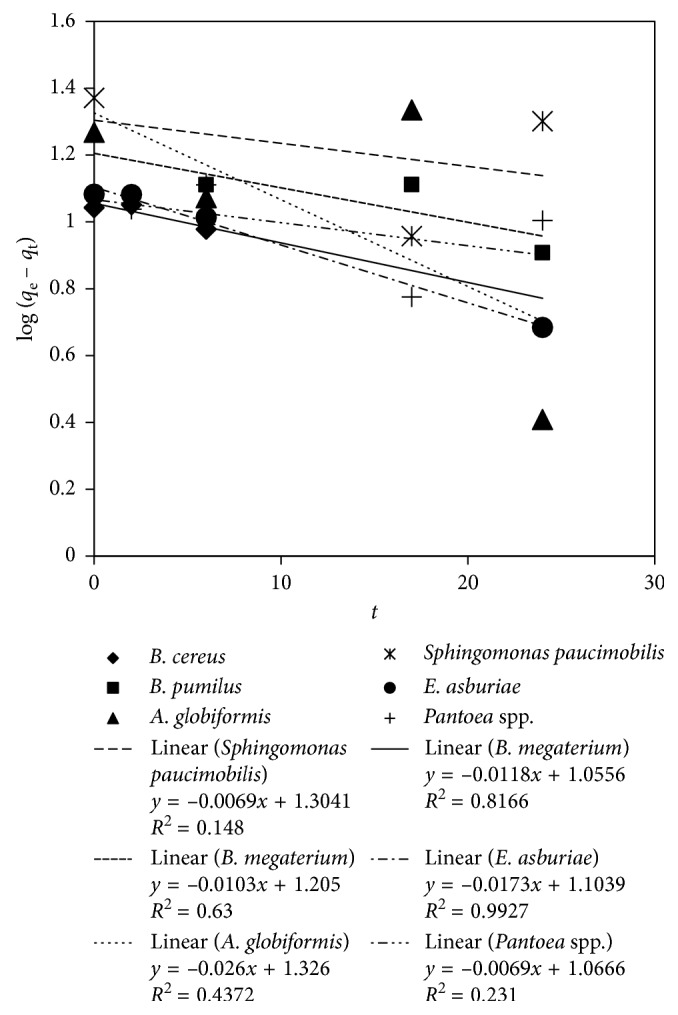
Pseudo-first-order of Lagergren plot for arsenic biosorption by the rhizobacteria.

Based on Figure 4, the pseudo-first-order model of Lagergren for arsenic biosorption of *E. asburiae* and *B. cereus* shows a high correlation coefficient (*R*^2^). The plot for both biosorbents resulted in an *R*^2^ of 0.82 (*B. cereus*) and 0.91 (*E. asburiae*). This finding indicates that the pseudo-first-order model of Lagergren best explains the arsenic biosorption of *E. asburiae* and *B. cereus*. The *q*_e_ and *k*_1_ obtained from the intercept and slope of equations (2) were 12.68 mg As/g biomass and 0.04/h for *E. asburiae* and 11.35 mg As/g biomass and 0.03/h for *B. cereus*. The other four rhizobacteria showed low *R*^2^ values. The summary of the pseudo-first-order of Lagergren kinetic model is listed in Table 2.

**Figure 5:**
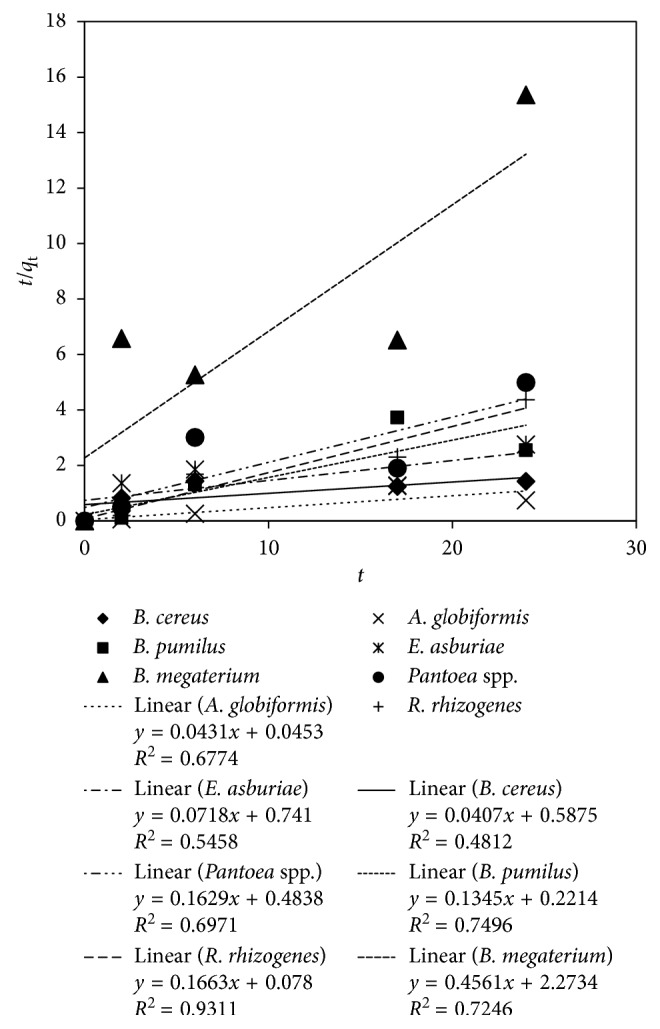
The pseudo-second-order plot for arsenic biosorption by the rhizobacteria.

Figure 5 demonstrates the pseudo-second-order plot for the seven rhizobacteria. The *R*^2^ of *R. rhizogenes* had the highest value (0.93), indicating that the pseudo-second-order model was favourable for the arsenic biosorption by *R. rhizogenes*. The obtained *q*_e_ was 6.02 mg As/g biomass, while the obtained *k*_2_ from the intercept was 0.5 mg As/g biomass/h (Table 2). The other rhizobacteria showed low *R*^2^ values.

**Figure 6:**
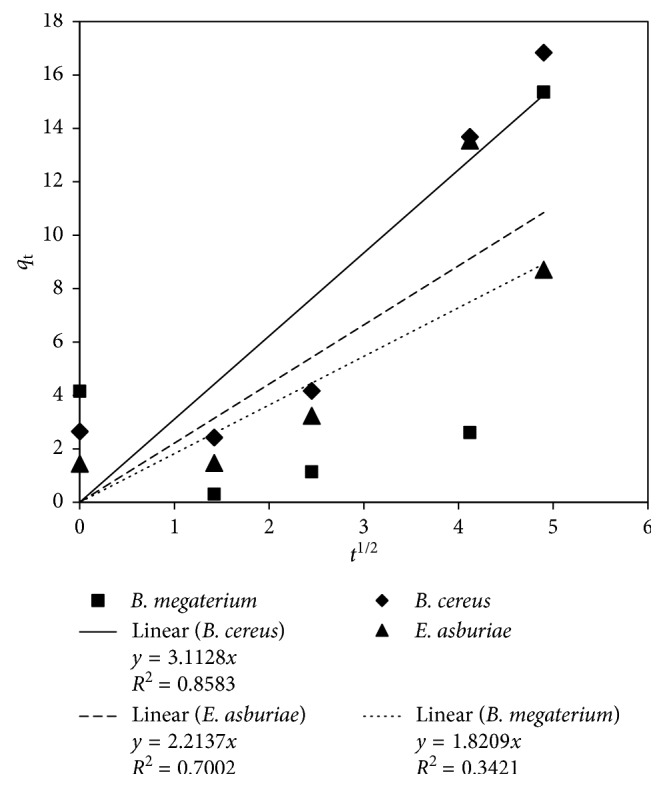
Pore diffusion plot for arsenic biosorption by the rhizobacteria.

**Figure 7:**
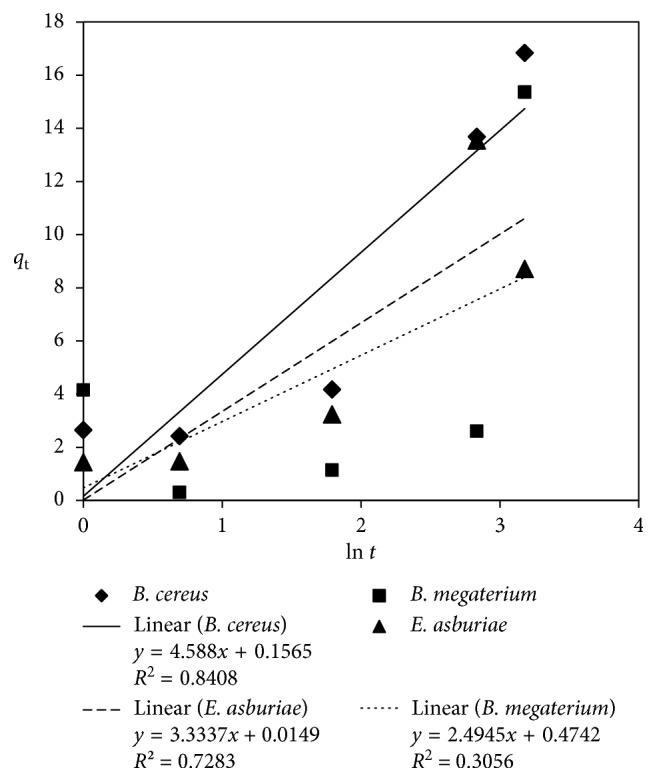
Elovich plot for arsenic biosorption by the rhizobacteria.

The *k*_d_ obtained from the slope of equation (6) was 3.11 mg As/g biomass/min^1/2^ for *B. cereus* (Figure 6). The pore diffusion model provided the highest *R*^2^ value for arsenic biosorption by *B. cereus* at 0.86. It indicates that the molecular diffusion of arsenic by *B. cereus* played an important role in the uptake capacity of *B. cereus*. The *R*^2^ of the Elovich kinetic model for *B. cereus* were much higher than the corresponding values of the other rhizobacteria (Figure 7).

The kinetic model of *B. cereus* could be calculated using the four models, but *R*^2^ was highest under the pore diffusion model. Meanwhile, the pseudo-first-order kinetic model for *E. asburiae* had the highest *R*^2^ value. The pseudo-second-order kinetic model for *R. rhizogenes* showed a higher *R*^2^ value than that of the other rhizobacteria. It can be concluded that each species of rhizobacteria has different kinetic models for arsenic biosorption.

## 4. Conclusions

Ten isolated rhizobacteria from *L. octovalvis* (*A. globiformis*, *B. megaterium, B. cereus, B. pumilus, Staphylococcus lentus, E. asburiae, Sphingomonas paucimobilis, Pantoea* spp., *R. rhizogenes, and R. radiobacter*) have different MICs after exposure to arsenate. *R. radiobacter* was the most resistant rhizobacteria to arsenate with a MIC >1500 mg/L. All rhizobacteria were able to biosorb arsenic. *Sphingomonas paucimobilis* showed a higher arsenic biosorption (146.4 ± 23.4 mg/g DCW) than the other nine rhizobacteria. All the resistant rhizobacteria have the potential as PGPR to enhance the arsenic phytoremediation. Based on the kinetic rate, *B. cereus, E. asburiae, and R. rhizogenes* had the highest *R*^2^ value of 0.86, 0.99, and 0.93 under the pore diffusion, pseudo-first-order kinetic, and pseudo-second-order kinetic models. It can be concluded that the MIC, arsenic biosorption capacity, and kinetic models in arsenic biosorption depend on the rhizobacterial species.

## Figures and Tables

**Table 1:** Summary of MIC on arsenate.

No.	Rhizobacteria	Gram stain	MIC (mg/L)
1	*B. megaterium*	+	250
2	*B. cereus*	+	250
3	*B. pumilus*	+	500
4	*A. globiformis*	+	500
5	*Staphylococcus lentus*	+	500
6	*Pantoea* spp.	−	250
7	*E. asburiae*	−	500
8	*Sphingomonas paucimobilis*	−	750
9	*R. rhizogenes*	−	750
10	*R. radiobacter*	−	>1500

**Table 2:** Kinetic constants for arsenic biosorption by rhizobacteria.

Rhizobacteria	Pseudo-first-order kinetic model			Pseudo-second-order kinetic model			Pore diffusion model		Elovich equation		
	*k*_1_ (h)	*q*_e_ (mg As/g biomass)	*R* ^2^	*k*_2_ (mg As/g biomass/h)	*q*_e_ (mg As/g biomass)	*R* ^2^	*k*_d_ (mg As/g biomass/h^1/2^)	*R* ^2^	*Α* (mg As/g biomass)	*β* (mg As/g biomass/h)	*R* ^2^
*B. cereus*	**0.03**	**11.35**	**0.82**	0.003	25.00	0.48	**3.11**	**0.86**	**4.82**	**0.22**	**0.84**
*B. pumilus*	0.02	13.20	0.63	0.08	7.46	0.75	—		—	—	
*B. megaterium*	—	—		0.09	2.19	0.72	1.82	0.34	2.62	0.40	0.31
*A. globiformis*	0.06	12.1	0.44	0.02	23.26	0.68	—		—	—	
*Staphylococcus lentus*	—	—		—	—		—		—	—	
*Sphingomonas paucimobilis*	0.01	20.14	0.15	—	—		—		—	—	
*E. asburiae*	**0.04**	**12.68**	**0.99**	0.01	14.08	0.55	2.21	0.70	3.50	0.30	0.73
*Pantoea* spp.	0.01	11.64	0.23	0.05	6.17	0.70	—		—	—	
*R. rhizogenes*	—	—		**0.35**	**6.02**	**0.93**	—		—	—	
*R. radiobacter*	—	—		—	—		—		—	—	

## Data Availability

The Excel sheet including the data used to support the findings of this study is available from the corresponding author upon request.
